# Quantitative contrast enhanced dual energy CT to predict avascular necrosis: a feasibility study of proximal humerus fractures

**DOI:** 10.1186/s12880-021-00717-x

**Published:** 2021-12-11

**Authors:** Kevin B. Hoover, Alexandria O. Starks, Valentina Robila, Daniel L. Riddle

**Affiliations:** 1 Mink Radiology, Cedars-Sinai Health System, 8670 Wilshire Blvd Suite 101, Beverly Hills, CA 90211 USA; 2 Orthopedic Associates of Lancaster, 170 North Pointe Blvd, Lancaster, PA 17601 USA; 3 Department of Pathology, Virginia Commonwealth University/VCU Health, 1101 East Marshall St, P.O. Box 980662, Richmond, VA 23298-0662 USA; 4 Otto D. Payton Professor of Physical Therapy, Orthopaedic Surgery and Rheumatology, Virginia Commonwealth University, Room B-100, West Hospital, 1200 East Broad Street, Richmond, VA 23298 USA

**Keywords:** Dual energy computed tomography, Iodine map, Avascular necrosis, Ischemia, Bone, Fracture

## Abstract

**Background:**

Avascular necrosis is a delayed complication of proximal humerus fractures that increases the likelihood of poor clinical outcomes. CT scans are routinely performed to guide proximal humerus fracture management. We hypothesized iodine concentration on post-contrast dual energy CT scans identifies subjects who develop avascular necrosis and ischemia due to compromised blood flow.

**Materials and methods:**

55 patients with proximal humerus fractures enrolled between 2014 and 2017 underwent clinical, radiographic and contrast enhanced dual energy CT assessment. Iodine densities of the humeral head and the glenoid (control) were measured on CT. Subjects managed with open reduction internal fixation or conservatively (non-surgical) were followed for up to two years for radiographic evidence of avascular necrosis. Arthroplasty subjects underwent histopathologic evaluation for ischemia of the resected humeral head.

**Results:**

17 of 55 subjects (30.9%) were treated conservatively, 21 (38.2%) underwent open reduction internal fixation and 17 of 55 (30.9%) underwent arthroplasty. Of the 38 subjects treated conservatively or with ORIF, 20 (52.6%) completed 12 months of follow up and 14 (36.8%) 24 months of follow up. At 12 months follow up, two of 20 subjects (10%) and at 24 months 3 of 14 subjects (21.4%) developed avascular necrosis. At 12 months, the mean humerus/glenoid iodine ratio was 1.05 (standard deviation 0.24) in subjects with AVN compared to 0.91 (0.24) in those who did not. At 24 months, subjects with avascular necrosis had a mean humerus/glenoid iodine concentration ratio of 1.06 (0.17) compared to 0.924 (0.21) in those who did not. Of 17 arthroplasty subjects, 2 had severe ischemia and an iodine ratio of 1.08 (0.30); 5 had focal ischemia and a ratio of 1.00 (0.36); and 8 no ischemia and a ratio of 0.83 (0.08).

**Conclusions:**

Quantifying iodine using dual energy CT in subjects with proximal humerus fractures is technically feasible. Preliminary data suggest higher humeral head iodine concentration may increase risk of avascular necrosis; however, future studies must enroll and follow enough subjects managed with open reduction internal fixation or conservatively for two or more years to provide statistically significant results.

*Trial Registrations* NCT02170545 registered June 23, 2014, ClinicalTrials.gov.

## Background

Displaced proximal humerus fractures represent a major challenge for patients and orthopedic surgeons. These fractures are the third most common type of long bone fracture after hip and distal radius fractures with approximately 20% undergoing surgical treatment [1–3]. One of the major fracture complications is avascular necrosis (AVN). This is secondary to impairment of the blood supply to the humeral head. This blood supply is primarily provided by the anterior and posterior circumflex humeral arteries and the anastomotic network between the two [4, 5]. The development of AVN is thought to occur in approximately 18% of displaced proximal humerus fractures within two years of injury. The incidence increases over time and estimates vary from 4 to 75% [6–9]. AVN may eventually lead to joint destruction with collapse of the articulating surface and is associated with poor patient outcomes [10–12].

Several of the clinical factors associated with humeral head fractures have been studied, but none have been shown to be predictive of future AVN using a prospective study design [13]. Greater fracture displacement and higher number of fracture fragments at the time of injury are associated with AVN [3, 14–16]. The relative risk of AVN from conservative and surgical treatment with open reduction internal fixation (ORIF) is controversial [13]. Efforts to predict AVN at the time of surgery based on pulsatile flow through surgical burr holes and laser doppler flowmetry have not been successful [7, 17].

Radiography and computed tomography (CT) are the current techniques of choice in the diagnosis and management of proximal humerus fractures [1, 3, 10]. Radiography is the first line imaging to assess fracture comminution, fracture displacement and glenohumeral joint dislocation. CT provides a more precise evaluation of fracture anatomy and joint alignment that is used in surgical planning [10]. Magnetic resonance imaging (MRI) is not commonly utilized in the evaluation of humeral head fractures.

MRI studies of femoral neck fractures demonstrate a decrease in contrast enhancement in subjects who developed AVN [18–22]. MRI, specifically dynamic contrast enhanced MRI, has shown promise in the prediction of AVN of the femoral head [18, 20–23]. However, this technique is an expensive, technically demanding and time intensive examination. It requires a high level of expertise to perform that may not be available at the time when patient management decisions are made. Furthermore, MRI is not routinely used in the post-operative setting due to the extensive artifact associated with metal from ORIF and arthroplasty hardware. For these and other reasons, MRI is not routinely used in either the surgical planning or the post-operative follow up of proximal humerus fractures.

In addition to the precise anatomic information available with CT, utilization of two kilovoltages, or dual energy (DECT), can be used to evaluate for gout, bone marrow edema and tissue perfusion [24–28]. DECT is a long-standing technology that has recently been commercialized and can distinguish different materials based on their X-ray attenuation at different voltages [26, 29, 30]. It can be used to distinguish trabecular bone from marrow constituents such as fat and collagen, calcium from iodinated contrast, different types of iodinated contrast and calcifications [25, 31–34]. This has led to an important clinical application: the virtual noncontrast reconstruction that removes iodinated contrast from contrast enhanced DECT images to distinguish contrast enhancement from calcium and blood. It also decreases the total radiation dosage to patients by eliminating the need for pre-contrast imaging [25, 26]. A contrast only reconstruction can be used to quantify the amount of iodine from intravenous contrast as a measure of tissue perfusion (i.e. iodine overlay or “iodine map”) [27, 30, 35]. Iodine maps have been primarily used to evaluate tumor vascularity before and after chemotherapy treatment [36, 37]. The use of DECT to measure blood perfusion of bone remains largely unexplored.

We hypothesized the quantity of iodine in the humeral head after intravenous contrast injection measured with DECT represents a surrogate marker of bone perfusion. Like the decrease in femoral head enhancement seen on MRI of femoral neck fractures, we hypothesized there would be decreased perfusion and iodine concentration in the humeral heads of subjects who have histopathologic evidence of ischemia and later develop radiographic evidence of AVN. To evaluate this hypothesis, we conducted a prognostic, prospective feasibility cohort study to measure the iodine concentration in the humeral head after fracture using iodine maps. Feasibility studies address areas that may impact the completion of a larger clinical study, including process, resources, management and scientific [38]. The purposes of our study were to determine: (1) if we could successfully recruit and retain subjects with proximal humerus fractures and follow those treated conservatively or with ORIF for two-years (process); (2) if we could identify subjects with histopathologic evidence of ischemia or radiographic evidence of AVN (scientific); (3) if iodine concentration measurements could be routinely obtained on study subjects (scientific); and (4) if there is preliminary evidence of iodine concentration differences in subjects with histopathologic evidence of ischemia or radiographic evidence of AVN (scientific).

## Methods

### Study design

This HIPAA compliant, prognostic, prospective feasibility cohort study of a newly proposed diagnostic test for quantifying AVN risk following proximal humerus fracture. was approved by the Institutional Review Board (IRB) at Virginia Commonwealth University. Patients 18 years and older with proximal humerus fractures were recruited during orthopedic inpatient consultations or office visits. Patients were excluded based on the following criteria: contralateral humerus AVN; history of iodinated intravenous (IV) contrast allergy with anaphylaxis; multiple myeloma due to additional risk of contrast induced kidney damage; sickle cell disease due to the pre-existing risk of AVN; pregnancy due to the risk of radiation to the fetus; and pre-existing renal insufficiency due to the additional risk of contrast induced nephropathy.

After signing an IRB approved consent form, enrolled subjects underwent a routine clinical evaluation, which includes a CT scan at our institution. Instead of a conventional CT scan using a single source and kVp, DECT of the affected shoulder was performed (see below). The subsequent clinical management was based on institutional usual care criteria such as clinical exam, radiographs and CT. While the surgeon was able to see the standard anatomic information provided by the CT scan, they were blinded to the quantitative data acquired from DECT (see below). As per clinical protocol, subjects were managed one of three ways: without surgical intervention (conservatively), with ORIF or by arthroplasty. Conservative management routinely utilized sling immobilization for two weeks followed by physical therapy. ORIF utilized a locked plate and screw construct with or without additional lag screws. Arthroplasty treatment utilized primarily anatomic components, but reverse arthroplasty components were also sometimes used depending on surgeon judgement and experience. Subjects were followed for a total of two years. This included a routine two-week postoperative visit and clinical visits every six months from the date of treatment plan (i.e., 6 months, 12 months, 18 months, and 24 months) that included proximal humerus radiographs to evaluate fracture union. Subjects received text and/or phone visit reminders prior to their scheduled appointment as all clinical patients receive at our institution. Subjects who did not attend scheduled follow up visits received follow up phone calls from clinic and study staff (KBH, AOS). Patient loss to follow up, or attrition, was registered by when it occurred in the six month cycle of appointments [39].

### Imaging

Radiographic evaluation of the fractured humerus included a minimum of two frontal views, with the humerus in internal and external rotation, and a lateral, scapular Y-view. A contrast enhanced DECT was also obtained prior to treatment using the dual source stellar detector Somatom Definition Flash (Siemens Healthcare) (Table 1). Delayed phase imaging was utilized. This was based on prior studies demonstrating bone enhancement and less enhancement variability than other phases [40, 41]. The anatomic CT data were used by all physicians managing the care of the study subjects. The source data were sent for post-processing analysis to Syngo.via (Siemens Healthcare).

**Table 1:** CT acquisition parameters

CT scanner	Somatom Definition Flash (Siemens Healthcare), dual source stellar detector
Contrast type/volume/injection rate/delay	Omnipaque 350/ 120 ml/ 3 ml/s /100 s
kVp	100 kVp and 140 kVp
Collimation/slice width/pitch	128 × 0.6 mm/3 mm/ 0.75
Kernels/increments/reformations	B30 (soft tissue) and B70 (bone)/3 mm/ bone and soft tissue axial, paracoronal and parasagittal 3 × 3 mm reformats using the mixed dataset

The liver virtual noncontrast application of the Syngo.Via was used to generate an iodine map to measure iodine concentration (mg/ml). Three areas of at least 1 cm^2^ were hand-drawn in three orthogonal planes (i.e., axial, paracoronal, parasagittal) within the subarticular trabecular bone of the humeral head by a radiologist with 12 year of subspecialized musculoskeletal radiology experience (KBH). The densities within these three areas were averaged to generate the humerus iodine concentration. Similarly, the iodine concentration of an area of at least 1 cm^2^ within the trabecular bone of the glenoid of the scapula (i.e., the glenoid vault), was measured in the three orthogonal planes and the densities averaged. The iodine concentration data were not available to physicians managing the care of the subjects.

Radiographs performed during follow up surgeon visits utilized at least two radiographic views, typically internal and external rotation views. The radiographs were assessed for imaging findings of AVN by the radiologist. Subjects were classified as having AVN if the humeral head demonstrated one or more of the following radiographic findings: a mixed lucent and sclerotic appearance, loss of sphericity and subchondral collapse (e.g., a “crescent sign”). If none of these findings were present during a 6-month follow-up examination the participant was coded as AVN negative for that visit.

### Histopathology

In subjects who underwent arthroplasty the humeral head was analyzed for histopathologic evidence of ischemia within 12 h of surgery. Upon gross evaluation of cartilage integrity and associated degenerative changes, the specimens were serially sectioned, in 3 mm thick slices, fixed in 10% buffered formalin for 12–24 h and decalcified using Decal solution (StatLab Medical Products, McKinney, TX). Sections were examined for presence of necrosis, thickened bone, or collapse of subchondral bone indicative of AVN. Microscopic parameters of ischemia were evaluated, including the presence of bone marrow necrosis and empty osteocytic lacunae. These findings were reported by the overall percentage of involvement. Based on the extent of ischemic change, the cases were classified as no ischemia, focal ischemia, or severe ischemia.

### Data analysis

Because we were interested in testing feasibility of the methods, our study was not powered for detecting differences in DECT values between those who did and did not develop AVN. Rather, our study was powered to detect problems with recruitment and loss to follow-up [42]. A sample of 50 persons gave us greater than 95% confidence in detecting a 20% loss to follow-up and a 20% rate of decline in patient consent.

Reporting of the results of this prospective cohort study followed the STROBE guidelines [43]. We report the characteristics of our sample using descriptive statistics and we compared those with and those without follow-up data to assess differences in the two populations. Additionally, we compare the demographic and clinical characteristics of the treatment groups and subjects without and with AVN using one-way analysis of variance (ANOVA), the Fischer Exact Test Chi-square and the Independent T-test using *p* ≤ 0.05 as a threshold for clinical significance and SAS software (SAS Institute Inc., Cary, NC, USA). Humerus iodine densities, glenoid iodine densities and humerus/glenoid iodine ratios for the three treatment categories are described and compared using ANOVA. Humerus iodine densities and humerus/glenoid iodine ratios in those with AVN and ischemia versus those without were described, but not statistically analyzed due to the low frequency of AVN.

## Results

### Enrollment and management

Sixty subjects provided informed written consent to participate in the study. Of these, 55 underwent a DECT scan (91.7%). The other five subjects underwent conventional, single energy CT for treatment planning and were excluded from the study. Of the 55 subjects, 30.9% were treated conservatively, 38.2% underwent open reduction internal fixation (ORIF) and 30.9% underwent arthroplasty (Table 2). The average age of subjects in the conservative treatment group was 63 years (SD 14), 46 years in the ORIF group (SD 16) and 71 years (SD 13) in the arthroplasty group. 10 female subjects were managed conservatively, 6 managed with ORIF, and 14 with arthroplasty.

**Table 2:** Subject characteristics by treatment group

Characteristics	Treatment			*p*
	Arthroplasty	Conservative	ORIF	
n	17	17	21	–
Age: Mean (SD)	71 (13)	63 (14)	46 (16)	< 0.001^a^
Gender	14 Female 3 Male	10 Female 7 Male	6 Female 15 Male	0.003^a^
Ethnicity	5 Black 1 Asian 11 White	6 Black 11 White	5 Black 16 White	NS^a^
*Iodine concentration mg/ml*				
Humerus (SD)	3.97 (0.70)	3.97 (0.97)	4.78 (1.22)	0.022^a^
Glenoid (SD)	4.51 (1.23)	4.72 (1.24)	5.17 (1.61)	NS^a^
Humerus/glenoid (SD)	0.94 (0.27)	0.88 (0.25)	0.96 (0.22)	NS^a^
*Follow up*				
12 Months		8	12	NS^b^
12 Months with AVN		0	2	NS^b^
24 Months		4	10	NS^b^
24 Months with AVN		0	3	NS^b^
*Fracture healing*				
Healed < 12 months		9	10	NS^b^
Healed ≤ 24 months		9	13	NS^b^

^a^One way ANOVA

^b^Fischer Exact Test Chi-square

The 38 subjects who were treated conservatively or by ORIF were followed clinically and radiographically for the development of AVN. 52.6% of the subjects completed 12 months of follow up and 36.8% of these completed a total of 24 months of follow up (Table 2). Post-hoc analysis of the demographics of study and subjects lost to follow up at 12 and 24 months indicates similar genders, ethnicities, medical conditions and numbers with steroid treatment with a higher age of subjects lost to follow up compared to experimental subjects at 24 months (Table 3). While the number of subjects was small, post-hoc analysis demonstrated no differences in the frequency of subjects managed conservatively or by ORIF who were lost to follow up (Table 2).

**Table 3:** Summary of comparisons between participants with and without follow-up data at 12 months and 24 months following recruitment

Characteristics	12 months			24 months		
	Study	Lost	*p*	Study	Lost	*p*
n	20	18		14	24	
Age (SD)	51 (17.6)	56 (17.3)	NS^a^	46.1 (17.3)	57.6 (16.3)	0.047^a^
Male	8	8	NS^b^	10	12	NS^b^
Female	12	10		4	12	
AA	3	8	NS^b^	2	9	NS^b^
White	17	10		12	15	
Diabetes Mellitus	4	3	NS^b^	1	6	NS^b^
Steroids	1	0	NS^b^	1	0	NS^b^

^a^Independent T-test

^b^Fischer Exact Test Chi-Square (2-sided)

### Fracture healing

Of the 17 patients treated conservatively 59% had radiographs during at least one of the follow up appointments (Table 2). All but one of these subjects healed their proximal humerus fractures over the 24-month trial period. The one subject who was incompletely healed at 6 months was lost to follow up.

Of the 21 subjects treated by ORIF, 71% subjects had radiographs during at least one of the follow up appointments. Of these 15 subjects, two did not heal their fractures. One was followed over two years and developed AVN. Another did not demonstrate a healed fracture after 6 months and was subsequently lost to follow up.

### Rate of avascular necrosis

**Fig. 1:**
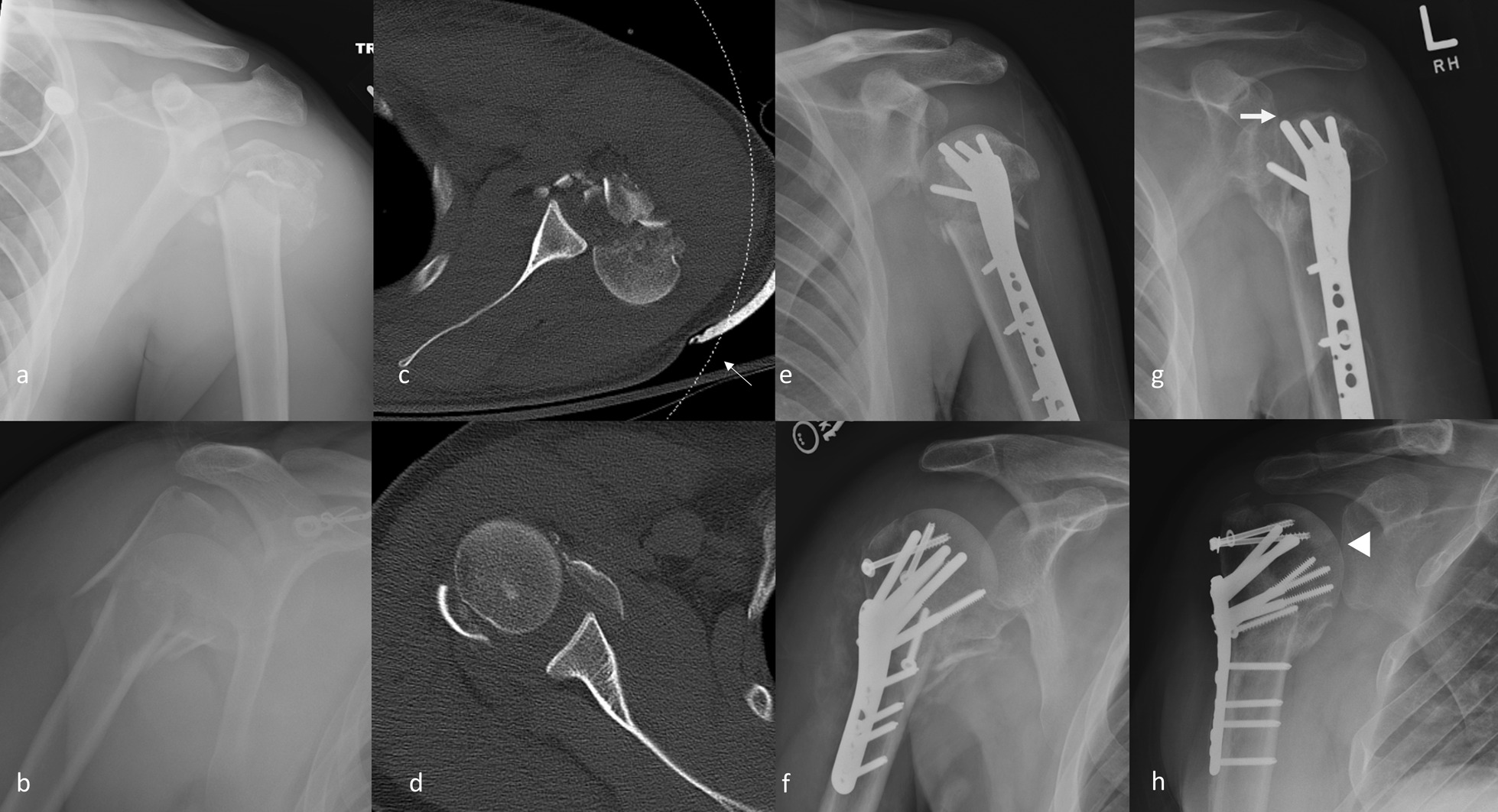
Initial radiographic and CT images with follow up imaging demonstrating AVN. Images from a 26-year-old male (top row, **a**, **c**, **e**, **g**) and a 36-year-old male (**b**, **d**, **f**, **h**) each with Neer four-part fractures were obtained including **a**, **b** initial frontal radiograph, **c**, **d** axial CT image, **e**, **f** post-ORIF radiograph and **g**, **h** evidence of osteonecrosis. **g** An attenuated linear shell of cortex with underlying lucency consistent with subchondral collapse due to AVN is present at 12 months after treatment (block arrow). **h** Subtle, circumscribed sclerosis adjacent to a focal lucency (block arrow) is consistent with AVN without collapse. The dashed line (**c**, thin arrow) indicates the outer margin of the 100 kVp source

Of the 20 subjects who had clinical and radiographic follow up for 12 months (Table 3), 10% had radiographic evidence of AVN (Fig. 1g–h). Of the 14 subjects who completed 24 months of follow up, 21.4% developed AVN. All subjects who developed AVN underwent ORIF following the fracture. Differences between the subjects without and with AVN were not studied using statistics due to the low number of subjects with AVN (Table 2).

### Iodine concentration and AVN

**Fig. 2:**
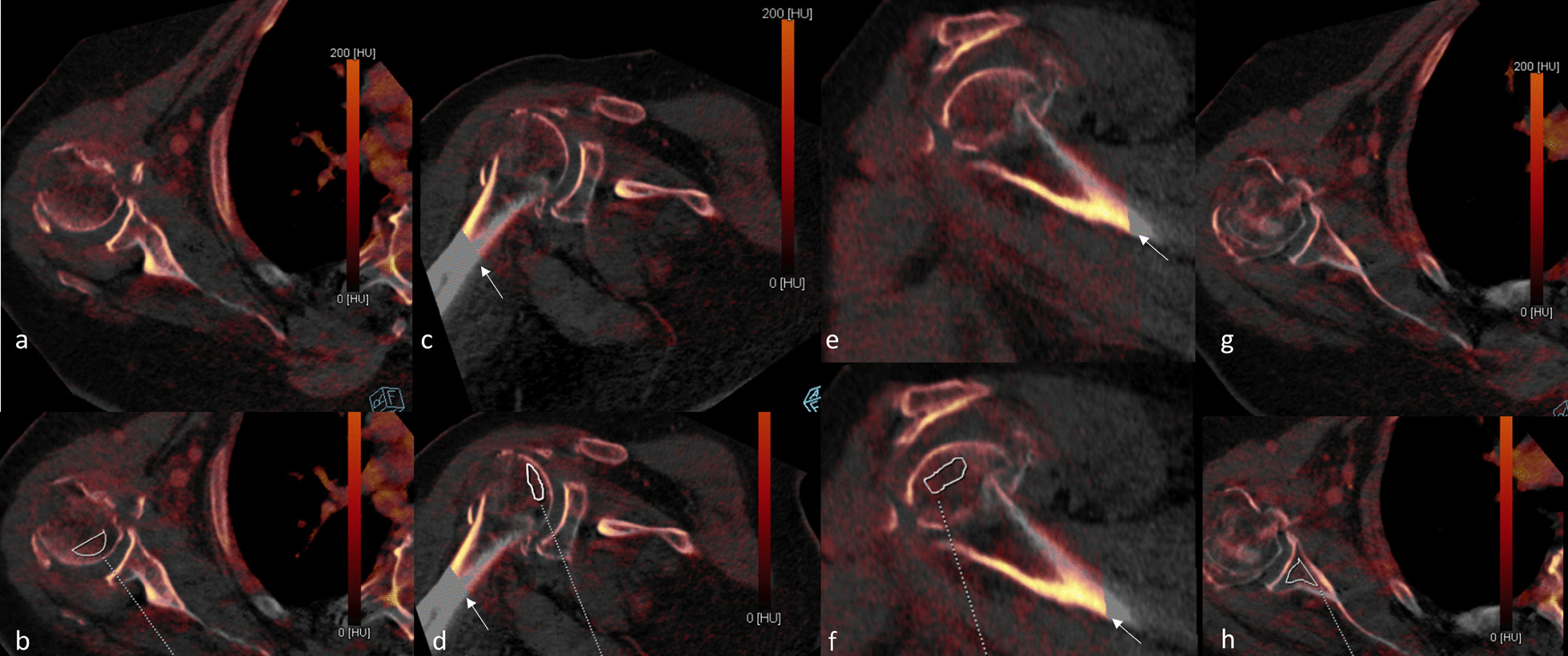
Iodine concentration measurements from DECT on an 86-year-old female subject with a Neer four-part proximal humerus fracture. The three planes of the right shoulder used for iodine concentration and humerus fragment measurements are shown: axial (**a**, **b**, **g**, **h**), coronal (**c**, **d**), sagittal (**e**, **f**). Areas were drawn in the humeral head fragment with overlying cartilage (**a**–**f**) and the glenoid vault (**g**, **h**) for iodine concentration measurement. The interfaces of the region with two energies (color, 100 and 140 kVp) and a single energy (no color, 140 kVp) is shown (thin arrows, **c**–**f**)

Iodine concentration measures were higher, on average, in subjects with AVN compared to those without AVN (Fig. 2, Table 4). Because of the small sample size of persons with AVN, results are described, but statistical significance was not evaluated. In the two subjects with AVN at 12 months, the average ratio of humeral head to glenoid iodine concentration (H/G ratio) was 15.4% higher in subjects with AVN. Iodine concentration in the humeral head was 20.2% higher in subjects with AVN. At 24 months, the H/G ratio was 14.7% higher in those with AVN and the humeral head iodine concentration was 7.8% higher in those with AVN.

**Table 4:** Study parameters in subjects with and without AVN at 12 months and 24 months

Iodine concentration (mg/ml)	12 months		24 months	
	AVN (n = 2)	No AVN (n = 18)	AVN (n = 3)	No AVN (n = 11)
Humerus (SD)	5.10 (1.46)	4.24 (1.35)	4.97 (1.06)	4.61 (1.46)
Glenoid (SD)	4.82 (0.31)	4.87 (1.76)	4.66 (.35)	5.08 (1.74)
Humerus/glenoid (SD)	1.05 (0.24)	0.91 (0.24)	1.06 (0.17)	0.92 (0.21)

Comparison of means was not performed due to the low number of subjects

### Ischemia

Humeral heads from 15 of 17 subjects who underwent shoulder arthroplasty underwent histopathologic analysis for parameters of ischemia (Table 5) [44–46]. The two subjects not analyzed either had only gross pathologic analysis or no pathology analysis. 46.7% of subjects had histopathologic evidence of ischemia (Table 5). Focal ischemia involving, on average, 20% of the tissue was identified in 33.3% of subjects. This presented as scattered areas of bone marrow necrosis associated with patchy empty osteocytic lacunae in the cortical bone. Severe ischemia involving approximately 30% of the tissue was present in 13.3% of specimens.

**Table 5:** Study parameters in subjects with and without focal ischemia and severe ischemia

Iodine concentration (mg/ml)	Focal ischemia (n = 5)	Severe ischemia (n = 2)	No ischemia (n = 8)
Humerus (SD)	4.19 (0.64)	4.47 (0.19)	3.85 (0.82)
Glenoid (SD)	4.57 (1.44)	4.27 (0.99)	4.66 (1.00)
Humerus/glenoid (SD)	1.00 (0.36)	1.08 (0.30)	0.83 (0.08)

Comparison of means was not performed due to the low number of subjects

The H/G ratios of subjects with severe ischemia and focal ischemia were 29.8% and 20.4% higher, respectively, than patients without ischemia (Table 5). The mean iodine concentration in subjects with severe ischemia and focal ischemia were 15.9% and 8.6% higher, respectively than those without ischemia. These values were not analyzed for statistical significance due to the small sample sizes.

## Discussion

This feasibility study of 55 subjects monitored for AVN successfully followed 53% of subjects for 12 months and 37% for 24 months. AVN was identified exclusively in subjects who underwent ORIF. This suggests that post-ORIF subjects should be followed for 2 years, or more, in the future study of AVN. While difficult to know how the number of subjects lost to follow up biased the study results, bias is likely [39, 47].

The incidence of AVN in the literature varies widely depending on fracture comminution and displacement, treatment, and duration of clinical follow up [13]. An incidence as high as 75% has been reported for the most comminuted fractures with nonoperative treatment followed for at least 24 months [48]. Lower incidences of AVN have been reported for ORIF subjects treated with plate and screw construct fixation: 8% at 12 months of follow up and 20% at 60 months years of follow up [6–8], which are similar to the 10% incidence at 12 months and 21.4% at 24 months in this study. An increase in incidence of AVN over time has previously been described in longitudinal studies comparing subjects at 12 months (4% of subjects) and 45 months (9%) and 35 months (26%) to 84 months (50%) [6, 9]. Unfortunately, there is no literature examining the histopathology of humerus fractures with which to compare our results.

Humerus to glenoid ratios of iodine concentration were elevated and similar in subjects with either AVN or ischemia at 12 and 24 months. While humeral head iodine concentrations were also elevated in AVN and ischemic subjects, they were higher at 12 months compared to 24 months. The similarity of the ratios at 12 and 24 months suggests this measurement may be more robust than the humerus iodine concentration alone.

Our results showing an increase in iodine concentration in AVN, and ischemia were the opposite of our initial hypothesis. This hypothesis was based on animal and human MRI studies of femoral neck fractures that showed a decrease in contrast enhancement including subjects who went on to develop AVN [18–22]. Animal studies indicate the disruption of arterial blood flow results in characteristic MRI changes and AVN. However, dynamic contrast enhanced MRI studies in subjects with atraumatic causes of AVN have demonstrated higher peak enhancement in femurs with AVN compared with normal adjacent bone and the normal contralateral femur [49–51]. The proposed mechanism for this relative increase in enhancement is vascular stasis due to venous outflow obstruction. Decreased venous outflow has been demonstrated in femoral head AVN subjects using intraosseous venography: an early diagnostic tool in the functional exploration of bone to determine the risk of AVN [52]. Perhaps differences in blood flow to the humeral and femoral heads help explain the greater iodine concentration measured in subjects with AVN.

Compared to the femoral head, the humeral head receives blood supply from multiple vessels. The femoral head is almost exclusively vascularized by a single vessel: the extracapsular medial circumflex artery. Increasing femur fracture comminution, displacement, and capsule injury is more likely to injure this vessel and compromise blood flow [23, 53, 54]. In contrast, the humeral head is perfused by both the anterior and posterior humeral circumflex arteries with intraosseous anastomoses [4, 5]. Specifically, in the area of the humeral head we measured, approximately 50% of blood flow is provided by each vessel [5]. Perhaps it is not arterial compromise that results in post-traumatic humerus AVN.

Time density analysis is an important next step to understand the reason for our results. Like the time intensity curves used in prior MRI studies, measurement of iodine density/concentration in humeral head fractures after contrast administration could confirm higher iodine concentration in subjects with AVN and elucidate the mechanism [18, 20–22, 49, 50]. Additional measurements of the axillary artery and vein along with the humeral head of human subjects at discrete time intervals could be performed on human subjects, similar to the MRI time intensity curves and measurement of CT brain perfusion used to evaluate strokes [55, 56].

This study has multiple strengths and weaknesses. One of the strengths was the novel and prospective use of CT technology. CT is already used routinely for the diagnosis of humerus fractures, but the use of IV contrast and dual energy CT to predict AVN has not been previously described. Conventional CT is hampered by the presence of calcium, which obscures the more subtle attenuation changes due to IV contrast. DECT allows the specific measurement of iodine concentration without the interference of calcium [33]. It also shows promise in determining tissue perfusion [28]. Recently, contrast enhanced DECT distinguished subjects with histopathologically proven AVN of the scaphoid bone from those without AVN [57]. In addition, contrast enhanced DECT was recently shown to detect enhancing bone metastasis, suggesting promise for the detection of areas of abnormal bone perfusion [58].

Another strength of this study was evaluation of compromised bone perfusion using two complementary techniques. Histopathologic evidence of early ischemia provides valuable early evidence of compromised perfusion. While less sensitive than other techniques in detection of AVN, radiographic evidence of AVN can provide specific evidence of decreased perfusion.

A significant weakness of this study was subject loss to follow up. The loss to follow up decreased the sample size and likely the number of subjects with AVN. The attrition of subjects was likely due to our reliance on clinical resources for follow up rather than the use of dedicated research coordinators and detailed follow up protocols and training [59]. Dedicated clinical research personnel is warranted in any future studies for better patient monitoring, communication and follow up. Another potential weakness was the use of radiography to determine the development of AVN. While this technique is not nearly as sensitive or specific as MRI or CT in the detection of AVN, it was chosen for multiple reasons. First, radiographs are routinely utilized in clinical settings to detect complications that warrant change in management, such as the identification of osteoarthritis that may indicate the need for shoulder arthroplasty. Second, while radiographs are relatively insensitive in detection of AVN, they allow excellent assessment of bone adjacent to hardware, such as collapse of the humeral head due to AVN and secondary osteoarthritis. This is clinically relevant, as patients with AVN may be asymptomatic [5]. MRI in the presence of hardware requires optimization including the use of time and expertise intensive protocols that have not been well described in the humerus [60]. Finally, radiography is relatively inexpensive compared to MRI, which was an important consideration for this pilot study.

Our feasibility study was designed, not for generalizability, but rather to inform the design of a larger study. If our DECT measures are representative, a two-group t-test with a 5% two-sided significance level will have 80% power to detect the difference between subjects without AVN (mean H/G ratio 0.92) from those who develop AVN (1.06) with a sample size of 106 subjects treated conservatively or with ORIF assuming a 20% incidence of AVN at two years. Fewer subjects might be necessary if follow-up is extended out to five years to increase the proportion of participants with AVN [6, 9]. While the role of ORIF in the development of AVN is controversial, based on our finding that only subjects treated with ORIF developed AVN, prospectively studying ORIF subjects may also decrease the total number of subjects required. The use of dedicated research staff to monitor subjects will be essential to reduce loss to follow up. Finally, the use of time concentration analysis in the next study of DECT will enhance our understanding of how iodine concentration may serve as an indicator of AVN risk in humeral head fractures.

## Conclusions

This feasibility study demonstrated the ability to successfully perform DECT with contrast on patients with humeral head fractures and provides useful baseline information to inform a larger study of the test to predict AVN. Further investigation of contrast enhanced DECT will better define its promise to predict those at increased risk of developing AVN.

## Data Availability

The datasets used and/or analyzed during the current study are available from the corresponding author on request.

## Abbreviations

ANOVA: Analysis of variance
AVN: Avascular necrosis
CT: Computed tomography
DECT: Dual energy computed tomography
IV: Intravenous
MRI: Magnetic resonance imaging
ORIF: Open reduction internal fixation
